# (2*R*,4*R*)-1-(*tert*-But­oxy­carbon­yl)-4-meth­oxy­pyrrolidine-2-carb­oxy­lic acid

**DOI:** 10.1107/S1600536810047707

**Published:** 2010-11-20

**Authors:** Jing Yuan, Zhi-Qiang Cai, Chang-Jiang Huang, Wei-Ren Xu

**Affiliations:** a Tianjin Key Laboratory of Molecular Drug Design and Discovery, Tianjin Institute of Pharmaceutical Research, Tianjin 300193, People’s Republic of China; b School of Pharmacy, Tianjin Medical University, Tianjin 300070, People’s Republic of China

## Abstract

In the title compound, C_11_H_19_NO_5_, the five-membered pyrrolidine ring adopts an envelope conformation. The dihedral angles between the carboxyl group plane, the pyrrolidine ring and the meth­oxy group are 59.50 (3) and 62.02 (1)°, respectively. In the crystal, inter­molecular O—H⋯O hydrogen bonds link the mol­ecules into chains along [100]. The absolute configuration is assigned in accord with that of (2*R*,4*R*)-1-(*tert*-but­oxy­carbon­yl)-4-hy­droxy­pyrrolidine-2-carb­oxy­lic acid, which was the starting material in the synthesis.

## Related literature

The title compound is an inter­mediate in the preparation of the direct FXa inhibitor, eribaxaban {systematic name: (2*R*,4*R*)-*N* ^1^-(4-chlorophenyl)-*N* ^2^-[2-fluoro-4-(2-oxopyridin-1(2*H*)-yl)phenyl]-4-methoxypyrrolidine-1,2-dicarboxamide}. For background to the bioactivity and applications of eribaxaban, see: Perzborn (2009 ▶); Kohrt *et al.* (2007 ▶). For the synthesis of other derivatives with proline, see: Van Huis *et al.* (2009 ▶); Bigge *et al.* (2003 ▶).
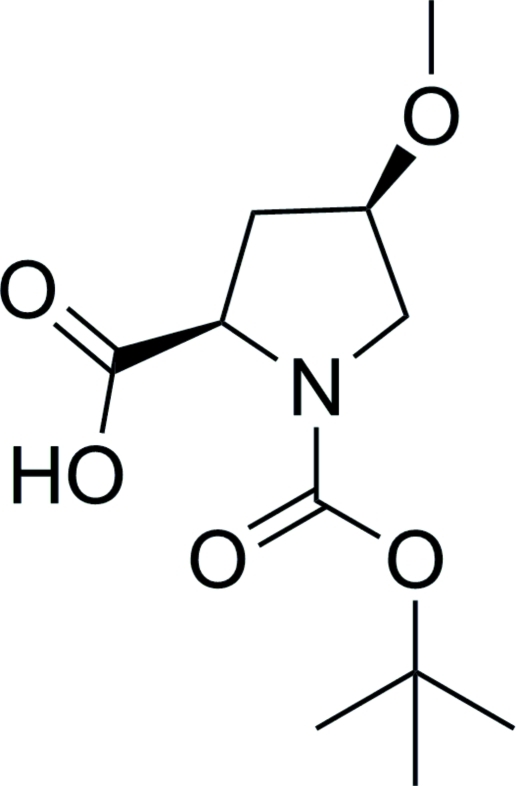


## Experimental

### 

#### Crystal data

C_11_H_19_NO_5_
*M* *_r_* = 245.27
Monoclinic, 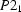

*a* = 6.4299 (13) Å
*b* = 9.784 (2) Å
*c* = 10.279 (2) Å
β = 90.12 (3)°
*V* = 646.7 (2) Å^3^
*Z* = 2
Mo *K*α radiation
μ = 0.10 mm^−1^
*T* = 293 K
0.26 × 0.20 × 0.10 mm

#### Data collection

Rigaku Saturn CCD area-detector diffractometer
Absorption correction: multi-scan (*CrystalClear*; Rigaku/MSC, 2005) ▶ *T*_min_ = 0.975, *T*_max_ = 0.990
7923 measured reflections
1601 independent reflections
1059 reflections with *I* > 2σ(*I*)
*R*_int_ = 0.059

#### Refinement

*R*[*F* ^2^ > 2σ(*F* ^2^)] = 0.037
*wR*(*F* ^2^) = 0.086
*S* = 0.94
1601 reflections
163 parameters
1 restraint
H atoms treated by a mixture of independent and constrained refinement
Δρ_max_ = 0.13 e Å^−3^
Δρ_min_ = −0.16 e Å^−3^

### 

Data collection: *CrystalClear* (Rigaku, 2005) ▶; cell refinement: *CrystalClear* ▶; data reduction: *CrystalClear* ▶; program(s) used to solve structure: *SHELXS97* (Sheldrick, 2008 ▶); program(s) used to refine structure: *SHELXL97* (Sheldrick, 2008 ▶); molecular graphics: *SHELXTL* (Sheldrick, 2008 ▶); software used to prepare material for publication: *SHELXTL*.

## Supplementary Material

Crystal structure: contains datablocks I, global. DOI: 10.1107/S1600536810047707/kp2285sup1.cif

Structure factors: contains datablocks I. DOI: 10.1107/S1600536810047707/kp2285Isup2.hkl

Additional supplementary materials:  crystallographic information; 3D view; checkCIF report

## Figures and Tables

**Table 1:** Hydrogen-bond geometry (Å, °)

*D*—H⋯*A*	*D*—H	H⋯*A*	*D*⋯*A*	*D*—H⋯*A*
O3—H3⋯O4^i^	1.00 (3)	1.68 (4)	2.672 (2)	169 (4)

Symmetry code: (i) 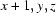.

**Fig. 1.**
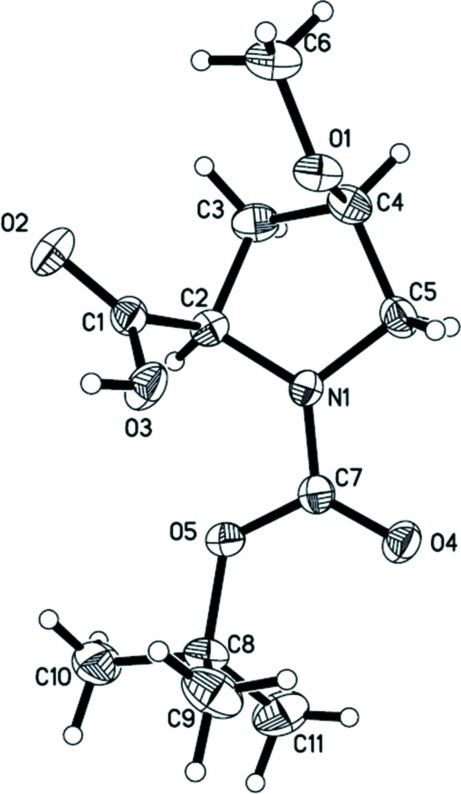
The structure of C11H19NO5 with all non-H atom-labelling scheme and ellipsoids drawn at the 50% probability level.

## supplementary crystallographic information

### Comment

Eribaxaban is a direct FXa inhibitors and has a high affinity for human FXa. Clinical data with eribaxaban in the prevention of VTE in TKR patients have recently been presented (Perzborn, 2009; Kohrt *et al.*, 2007).

The title compound (Fig. 1), (2*R*,4*R*)-1-(*tert*-butoxycarbonyl)-4-methoxypyrrolidine-2-carboxylic acid is important intermediate in the preparation of Eribaxaban. Some derivatives of Eribaxaban have been reported with high affinity for human FXa (Van Huis *et al.*, 2009; Bigge *et al.*, 2003). Herein, the synthesis and the crystal structure of the title compound are reported; the absolute configuration is assigned by the use of (2*R*,4*R*)-1-(*tert*-butoxycarbonyl)-4- hydroxypyrrolidine-2-carboxylic acid as the starting material for the synthesis.

The pyrrolidine ring of the title compound adopts an envelope conformation with the C3 lying out of the plane. The dihedral angles between the carboxyl group plane, pyrrolidine ring and methoxy system are 120.50 (3)° and 117.98 (1)°, respectively. In the crystal structure, intermolecular O—H···O interactions contribute to the stabilization of the packing. Each molecule is a donor and acceptor for 2 hydrogen bonds (TAble 1).

### Experimental

CH_3_I (22 g, 0.155 mol) and 60%(*w*/*w*)NaH (15 g, 0.625 mol) were dissolved in THF(300 ml), and the resulting mixture was cooled to 273 K in an ice bath. (*R*,*R*)-4-Hydroxy-pyrrolidine-1,2-dicarboxylic acid, 1-*tert*-butyl ester(35 g, 0.151 mol)was then added in portions while maintaining a reaction temperature of 278 K or less. The reaction was allowed to warm to 293 K overnight. To the reaction mixture was added H2O (100 ml), 1 N HCl(100 ml) and NaCl(42 g). The layers were separated, and the organic layer was dried over MgSO4, filtered and concentrated to the white solid (37 g). Colourless single crystals suitable for X-ray diffraction were obtained by recrystallisation from methanol.

### Refinement

All H atoms were geometrically positioned (C—H 0.93–0.98 Å) and treated as riding, with *U*_iso_(H) = 1.2Ueq(C).

### Crystal data

**Table:**

C_11_H_19_NO_5_	*F*(000) = 264
*M**_r_* = 245.27	*D*_x_ = 1.260 Mg m^−^^3^
Monoclinic, *P*2_1_	Mo *K*α radiation, λ = 0.71073 Å
Hall symbol: P2yb	Cell parameters from 1953 reflections
*a* = 6.4299 (13) Å	θ = 3.2–27.8°
*b* = 9.784 (2) Å	µ = 0.10 mm^−^^1^
*c* = 10.279 (2) Å	*T* = 293 K
β = 90.12 (3)°	Prism, colorless
*V* = 646.7 (2) Å^3^	0.26 × 0.20 × 0.10 mm
*Z* = 2	

### Data collection

**Table:**

Rigaku Saturn CCD area-detector diffractometer	1601 independent reflections
Radiation source: rotating anode	1059 reflections with *I* > 2σ(*I*)
confocal	*R*_int_ = 0.059
ω and φ scans	θ_max_ = 27.7°, θ_min_ = 3.2°
Absorption correction: multi-scan (*CrystalClear*; Rigaku/MSC, 2005)	*h* = −8→8
*T*_min_ = 0.975, *T*_max_ = 0.990	*k* = −12→12
7923 measured reflections	*l* = −13→13

### Refinement

**Table:**

Refinement on *F*^2^	Secondary atom site location: difference Fourier map
Least-squares matrix: full	Hydrogen site location: inferred from neighbouring sites
*R*[*F*^2^ > 2σ(*F*^2^)] = 0.037	H atoms treated by a mixture of independent and constrained refinement
*wR*(*F*^2^) = 0.086	*w* = 1/[σ^2^(*F*_o_^2^) + (0.0494*P*)^2^] where *P* = (*F*_o_^2^ + 2*F*_c_^2^)/3
*S* = 0.94	(Δ/σ)_max_ < 0.001
1601 reflections	Δρ_max_ = 0.13 e Å^−^^3^
163 parameters	Δρ_min_ = −0.16 e Å^−^^3^
1 restraint	Extinction correction: *SHELXL97* (Sheldrick, 2008), Fc^*^=kFc[1+0.001xFc^2^λ^3^/sin(2θ)]^-1/4^
Primary atom site location: structure-invariant direct methods	Extinction coefficient: 0.55 (4)

### Special details

**Table:**

Geometry. All e.s.d.'s (except the e.s.d. in the dihedral angle between two l.s. planes) are estimated using the full covariance matrix. The cell e.s.d.'s are taken into account individually in the estimation of e.s.d.'s in distances, angles and torsion angles; correlations between e.s.d.'s in cell parameters are only used when they are defined by crystal symmetry. An approximate (isotropic) treatment of cell e.s.d.'s is used for estimating e.s.d.'s involving l.s. planes.
Refinement. Refinement of *F*^2^ against ALL reflections. The weighted *R*-factor *wR* and goodness of fit *S* are based on *F*^2^, conventional *R*-factors *R* are based on *F*, with *F* set to zero for negative *F*^2^. The threshold expression of *F*^2^ > σ(*F*^2^) is used only for calculating *R*-factors(gt) *etc*. and is not relevant to the choice of reflections for refinement. *R*-factors based on *F*^2^ are statistically about twice as large as those based on *F*, and *R*- factors based on ALL data will be even larger.

### Fractional atomic coordinates and isotropic or equivalent isotropic displacement parameters (Å^2^)

**Table:**

	*x*	*y*	*z*	*U*_iso_*/*U*_eq_	
O1	0.9616 (2)	0.74085 (17)	0.56702 (14)	0.0482 (4)	
O2	1.2349 (3)	0.63017 (18)	0.30520 (17)	0.0550 (5)	
O3	1.1022 (3)	0.83878 (17)	0.27707 (17)	0.0505 (5)	
H3	1.253 (5)	0.861 (4)	0.262 (3)	0.093 (11)*	
O4	0.4931 (2)	0.92660 (17)	0.24970 (16)	0.0472 (5)	
O5	0.6949 (2)	0.80182 (16)	0.10995 (13)	0.0462 (5)	
N1	0.7106 (2)	0.7625 (2)	0.32240 (16)	0.0387 (4)	
C1	1.0873 (3)	0.7059 (2)	0.2972 (2)	0.0383 (5)	
C2	0.8634 (3)	0.6534 (2)	0.3036 (2)	0.0375 (5)	
H2	0.8304	0.6019	0.2244	0.045*	
C3	0.8259 (4)	0.5636 (2)	0.4235 (2)	0.0490 (6)	
H3A	0.7087	0.5027	0.4103	0.059*	
H3B	0.9482	0.5099	0.4448	0.059*	
C4	0.7804 (4)	0.6680 (2)	0.5283 (2)	0.0461 (6)	
H4	0.7092	0.6269	0.6028	0.055*	
C5	0.6428 (3)	0.7725 (3)	0.45841 (19)	0.0454 (6)	
H5A	0.4968	0.7493	0.4670	0.054*	
H5B	0.6654	0.8638	0.4924	0.054*	
C6	1.1031 (4)	0.6610 (3)	0.6413 (2)	0.0632 (8)	
H6A	1.0289	0.6135	0.7084	0.095*	
H6B	1.2057	0.7197	0.6801	0.095*	
H6C	1.1706	0.5960	0.5856	0.095*	
C7	0.6240 (3)	0.8376 (2)	0.2284 (2)	0.0381 (5)	
C8	0.6706 (4)	0.8926 (2)	−0.0042 (2)	0.0463 (6)	
C9	0.7760 (5)	1.0273 (3)	0.0251 (3)	0.0712 (9)	
H9A	0.7098	1.0697	0.0983	0.107*	
H9B	0.7650	1.0863	−0.0493	0.107*	
H9C	0.9200	1.0115	0.0447	0.107*	
C10	0.7858 (5)	0.8146 (4)	−0.1077 (2)	0.0734 (9)	
H10A	0.9256	0.7975	−0.0791	0.110*	
H10B	0.7884	0.8672	−0.1865	0.110*	
H10C	0.7167	0.7291	−0.1235	0.110*	
C11	0.4450 (5)	0.9100 (4)	−0.0410 (3)	0.0750 (9)	
H11A	0.3765	0.8229	−0.0382	0.113*	
H11B	0.4356	0.9469	−0.1274	0.113*	
H11C	0.3794	0.9714	0.0191	0.113*	

### Atomic displacement parameters (Å^2^)

**Table:**

	*U*^11^	*U*^22^	*U*^33^	*U*^12^	*U*^13^	*U*^23^
O1	0.0579 (10)	0.0464 (10)	0.0402 (8)	−0.0032 (8)	−0.0090 (7)	0.0051 (7)
O2	0.0423 (9)	0.0491 (10)	0.0735 (11)	0.0155 (9)	−0.0065 (7)	−0.0084 (8)
O3	0.0326 (9)	0.0405 (10)	0.0783 (12)	0.0026 (8)	0.0053 (7)	0.0105 (9)
O4	0.0349 (9)	0.0452 (10)	0.0614 (10)	0.0048 (8)	0.0058 (7)	0.0067 (7)
O5	0.0514 (9)	0.0497 (10)	0.0374 (9)	0.0110 (8)	−0.0016 (6)	0.0051 (7)
N1	0.0300 (9)	0.0469 (11)	0.0393 (10)	0.0032 (9)	0.0017 (7)	0.0045 (8)
C1	0.0368 (13)	0.0415 (13)	0.0364 (11)	0.0045 (10)	−0.0013 (9)	−0.0039 (9)
C2	0.0378 (11)	0.0365 (12)	0.0384 (11)	−0.0004 (10)	−0.0062 (8)	−0.0033 (9)
C3	0.0561 (16)	0.0360 (13)	0.0548 (14)	−0.0073 (11)	−0.0064 (11)	0.0074 (10)
C4	0.0509 (14)	0.0451 (15)	0.0424 (12)	−0.0089 (11)	0.0027 (10)	0.0084 (10)
C5	0.0411 (12)	0.0557 (15)	0.0393 (12)	−0.0021 (12)	0.0076 (9)	0.0029 (11)
C6	0.0698 (17)	0.073 (2)	0.0469 (15)	0.0031 (16)	−0.0158 (12)	0.0085 (13)
C7	0.0270 (10)	0.0422 (13)	0.0451 (12)	−0.0051 (10)	0.0012 (9)	0.0042 (10)
C8	0.0563 (15)	0.0457 (14)	0.0367 (12)	−0.0018 (11)	−0.0070 (10)	0.0064 (9)
C9	0.100 (2)	0.0617 (19)	0.0519 (16)	−0.0284 (18)	0.0041 (15)	0.0033 (13)
C10	0.103 (2)	0.076 (2)	0.0412 (14)	0.0196 (19)	0.0010 (14)	0.0007 (14)
C11	0.072 (2)	0.076 (2)	0.078 (2)	0.0082 (17)	−0.0301 (15)	0.0039 (16)

### Geometric parameters (Å, °)

**Table:**

O1—C6	1.421 (3)	C4—H4	0.9800
O1—C4	1.422 (3)	C5—H5A	0.9700
O2—C1	1.206 (3)	C5—H5B	0.9700
O3—C1	1.320 (3)	C6—H6A	0.9600
O3—H3	1.00 (3)	C6—H6B	0.9600
O4—C7	1.231 (3)	C6—H6C	0.9600
O5—C7	1.347 (3)	C8—C10	1.506 (3)
O5—C8	1.480 (3)	C8—C11	1.508 (4)
N1—C7	1.335 (3)	C8—C9	1.512 (4)
N1—C2	1.464 (3)	C9—H9A	0.9600
N1—C5	1.469 (3)	C9—H9B	0.9600
C1—C2	1.530 (3)	C9—H9C	0.9600
C2—C3	1.533 (3)	C10—H10A	0.9600
C2—H2	0.9800	C10—H10B	0.9600
C3—C4	1.513 (3)	C10—H10C	0.9600
C3—H3A	0.9700	C11—H11A	0.9600
C3—H3B	0.9700	C11—H11B	0.9600
C4—C5	1.530 (3)	C11—H11C	0.9600
			
C6—O1—C4	113.5 (2)	O1—C6—H6A	109.5
C1—O3—H3	108 (2)	O1—C6—H6B	109.5
C7—O5—C8	121.67 (18)	H6A—C6—H6B	109.5
C7—N1—C2	125.79 (17)	O1—C6—H6C	109.5
C7—N1—C5	121.89 (19)	H6A—C6—H6C	109.5
C2—N1—C5	112.03 (17)	H6B—C6—H6C	109.5
O2—C1—O3	123.9 (2)	O4—C7—N1	123.01 (19)
O2—C1—C2	122.1 (2)	O4—C7—O5	125.3 (2)
O3—C1—C2	113.93 (19)	N1—C7—O5	111.70 (19)
N1—C2—C1	113.13 (18)	O5—C8—C10	101.8 (2)
N1—C2—C3	101.81 (17)	O5—C8—C11	111.5 (2)
C1—C2—C3	112.11 (17)	C10—C8—C11	110.7 (2)
N1—C2—H2	109.8	O5—C8—C9	108.62 (19)
C1—C2—H2	109.8	C10—C8—C9	111.2 (2)
C3—C2—H2	109.8	C11—C8—C9	112.5 (2)
C4—C3—C2	102.49 (18)	C8—C9—H9A	109.5
C4—C3—H3A	111.3	C8—C9—H9B	109.5
C2—C3—H3A	111.3	H9A—C9—H9B	109.5
C4—C3—H3B	111.3	C8—C9—H9C	109.5
C2—C3—H3B	111.3	H9A—C9—H9C	109.5
H3A—C3—H3B	109.2	H9B—C9—H9C	109.5
O1—C4—C3	112.24 (19)	C8—C10—H10A	109.5
O1—C4—C5	105.62 (19)	C8—C10—H10B	109.5
C3—C4—C5	103.28 (17)	H10A—C10—H10B	109.5
O1—C4—H4	111.7	C8—C10—H10C	109.5
C3—C4—H4	111.7	H10A—C10—H10C	109.5
C5—C4—H4	111.7	H10B—C10—H10C	109.5
N1—C5—C4	103.27 (19)	C8—C11—H11A	109.5
N1—C5—H5A	111.1	C8—C11—H11B	109.5
C4—C5—H5A	111.1	H11A—C11—H11B	109.5
N1—C5—H5B	111.1	C8—C11—H11C	109.5
C4—C5—H5B	111.1	H11A—C11—H11C	109.5
H5A—C5—H5B	109.1	H11B—C11—H11C	109.5
			
C7—N1—C2—C1	85.8 (2)	C7—N1—C5—C4	179.02 (19)
C5—N1—C2—C1	−100.3 (2)	C2—N1—C5—C4	4.8 (2)
C7—N1—C2—C3	−153.7 (2)	O1—C4—C5—N1	89.6 (2)
C5—N1—C2—C3	20.2 (2)	C3—C4—C5—N1	−28.4 (2)
O2—C1—C2—N1	166.64 (19)	C2—N1—C7—O4	178.7 (2)
O3—C1—C2—N1	−16.0 (2)	C5—N1—C7—O4	5.3 (3)
O2—C1—C2—C3	52.2 (3)	C2—N1—C7—O5	−0.3 (3)
O3—C1—C2—C3	−130.4 (2)	C5—N1—C7—O5	−173.6 (2)
N1—C2—C3—C4	−37.2 (2)	C8—O5—C7—O4	18.7 (3)
C1—C2—C3—C4	84.0 (2)	C8—O5—C7—N1	−162.41 (18)
C6—O1—C4—C3	−71.9 (2)	C7—O5—C8—C10	175.9 (2)
C6—O1—C4—C5	176.29 (19)	C7—O5—C8—C11	−65.9 (3)
C2—C3—C4—O1	−72.3 (2)	C7—O5—C8—C9	58.5 (3)
C2—C3—C4—C5	40.9 (2)		

### Hydrogen-bond geometry (Å, °)

**Table:**

*D*—H···*A*	*D*—H	H···*A*	*D*···*A*	*D*—H···*A*
O3—H3···O4^i^	1.00 (3)	1.68 (4)	2.672 (2)	169 (4)

Symmetry codes: (i) *x*+1, *y*, *z*.
